# A Novel IDS with a Dynamic Access Control Algorithm to Detect and Defend Intrusion at IoT Nodes

**DOI:** 10.3390/s24072188

**Published:** 2024-03-29

**Authors:** Moutaz Alazab, Albara Awajan, Hadeel Alazzam, Mohammad Wedyan, Bandar Alshawi, Ryan Alturki

**Affiliations:** 1 Department of Intelligent Systems, Faculty of Artificial Intelligence, Al-Balqa Applied University, Al-Salt 19385, Jordan; a.awajan@bau.edu.jo (A.A.); hadeel.alazzam@bau.edu.jo (H.A.); 2 Cybersecurity Department, School of Computing and Data Sciences, Oryx Universal College with Liverpool John Moores University, Doha 34110, Qatar; 3 Department of Computer Sciences, Faculty of Information Technology and Computer Sciences, Yarmouk University (YU), Irbid 21163, Jordan; mwedyan@yu.edu.jo; 4 Department of Computer and Network Engineering, College of Computing, Umm Al-Qura University, Makkah 24382, Saudi Arabia; bmhshawi@uqu.edu.sa; 5 Department of Software Engineering, College of Computing, Umm Al-Qura University, Makkah 24382, Saudi Arabia; rmturki@uqu.edu.sa

**Keywords:** Intrusion Detection System, LSTM, deep learning, detection rate, optimization, Dynamic Access Control

## Abstract

The Internet of Things (IoT) is the underlying technology that has enabled connecting daily apparatus to the Internet and enjoying the facilities of smart services. IoT marketing is experiencing an impressive 16.7% growth rate and is a nearly USD 300.3 billion market. These eye-catching figures have made it an attractive playground for cybercriminals. IoT devices are built using resource-constrained architecture to offer compact sizes and competitive prices. As a result, integrating sophisticated cybersecurity features is beyond the scope of the computational capabilities of IoT. All of these have contributed to a surge in IoT intrusion. This paper presents an LSTM-based Intrusion Detection System (IDS) with a Dynamic Access Control (DAC) algorithm that not only detects but also defends against intrusion. This novel approach has achieved an impressive 97.16% validation accuracy. Unlike most of the IDSs, the model of the proposed IDS has been selected and optimized through mathematical analysis. Additionally, it boasts the ability to identify a wider range of threats (14 to be exact) compared to other IDS solutions, translating to enhanced security. Furthermore, it has been fine-tuned to strike a balance between accurately flagging threats and minimizing false alarms. Its impressive performance metrics (precision, recall, and F1 score all hovering around 97%) showcase the potential of this innovative IDS to elevate IoT security. The proposed IDS boasts an impressive detection rate, exceeding 98%. This high accuracy instills confidence in its reliability. Furthermore, its lightning-fast response time, averaging under 1.2 s, positions it among the fastest intrusion detection systems available.

## 1. Introduction

The Internet of Things (IoT) is the technology between machines and the internet, enabling multiple facilities, including ubiquitous control, monitoring, and smart services [1]. The advent of the 5G network and the availability of high-speed internet access has fueled the growth of this sector [2]. At the current growth rate of 16.7% [3], the net IoT market worth is expected to cross USD 300 billion [4]. However, this massive market suffers from security vulnerabilities. IoT devices are compact, low-cost, and consume little power. With these features in an IoT product, a company will lose market competition. These characteristics limit the scope of incorporating advanced cybersecurity features into IoT devices [5]. The accelerated growth, the massive worth of the market, the reward of accessing interconnected nodes, and existing vulnerabilities make IoT an attractive ground for cybercriminals. This paper presents a practical IoT intrusion detection system that has been developed, keeping utility and usability at the center of the design. This innovative and practical IDS with novel features is a potential solution to IoT intrusions.

The methodology proposed in this paper has been developed to address the research gap discovered in the literature review presented in this paper. The effectiveness of the deep learning (DL) approaches in different papers testifies to the feasibility of developing a practical IDS [6]. In this endeavor, the first challenge is selecting an effective DL model, which is done in this paper through mathematical characteristics analysis [7]. Unlike most of the deep learning-based IDS research, this paper does not jump to any conclusions with the development of the technology only. An application model has been developed and presented in this paper to apply the proposed IDS. It has been achieved by developing an algorithm that dynamically controls access to IoT devices based on the network traffic level of maliciousness. It senses the malicious signals and blocks the malicious traffic source for a period of time, which depends on the number of intrusion attempts performed by the source. This unique feature makes the proposed IDS a practical solution to strengthen IoT security.

Furthermore, this paper observes IDS development from a utility and usability perspective. It is common for machine learning approaches to get some false positive (FP) predictions. Responding to every prediction and blocking the traffic based on false positive predictions undermine the utility offered by any IDS [8]. Moreover, it severely impacts the usability of the IDS. These product-centric characteristics are absent in most IDS research because of the lack of a proper application model. The proposed IDS introduces an innovative analysis approach that balances detection and false positive rates. Although this optimization technique marginally reduces the detection rate, it improves the user experience by reducing the service interruption caused by false alarms. As a result, the proposed IDS is an excellent solution from a utility and usability perspective.

This paper selects the DL network through mathematical analysis, which ensures better and more reliable performance with justification. Instead of using an existing network, this paper has presented an innovative approach to optimizing network architecture for intrusion detection. Integrating the Dynamic Access Control (DAC) algorithm is one of this paper’s novel contributions, extending the capability of the proposed IDS from detection to defense. It dynamically blocks the malicious traffic source for periods of time, depending on the intrusion volume. Another novelty of this paper is identifying the optimization issue of false positive and detection rates and solving it through an appropriate threshold selection method. The unique features and novel contributions of this paper are listed below:

- Development of the novel Dynamic Access Control (DAC) algorithm for defending against further intrusion from the same source by blocking it for periods associated with the number of intrusions.
- Applying the unique concept of detection rate optimization to enhance the usability of intrusion detection systems by minimizing the false positive rate, which lowers the probability of service interruption for false alarms.
- Achieving 97.16% validation accuracy, with precision, recall, and F1 score of 97.07%, 97.11%, and 97.05%, respectively.
- Gaining a detection rate of 98.73% by maintaining an average detection time of 1.14 s, which is considered to be real-time detection.

The rest of the paper has been organized into six sections. Section 2 presents a review of the recent literature. The methodology proposed in this paper is meticulously detailed in Section 3. Section 4 outlines the specific implementation and response mechanisms of the proposed Intrusion Detection System (IDS). The findings from the experiments and an analysis of their effectiveness are presented in Section 5. Section 6 highlights the limitations of the paper and discusses potential avenues for the future direction of this research. Finally, Section 7 concludes the paper by summarizing it.

## 2. Literature Review

The study by A. Awajan [9] on DL application achieves 93.74% accuracy for five frequently occurring IoT intrusions. The fully connected (FC) network architecture presented in this paper is incapable of interpreting network sequential data, which is a significant disadvantage of this paper. D. Musleh et al. applied machine learning (ML) to extract features and detect intrusion from image data. While their performance is impressive, the practical application of it is limited because it considers image features only [10]. A similar ML-based study conducted by S. Alkadi et al. [11] analyzes six ML models on four datasets related to IoT intrusion. While this paper provides deep insights into ML applications in IDS research, it does not contribute to developing any practical solution. An interesting study by M. Alazab et al. [12] applies the Moth–Flame Optimizer (MFO) algorithm for intrusion detection. While this approach deserves appreciation for impressive performance, it does not discuss the effective application of the performance. Unlike these approaches, the proposed IDS has been developed from an application perspective. That is why detection and defending have received equal attention in this paper, which is absent in this literature.

R. Chaganti et al. [13] have proposed an IDS for a software-defined networking (SDN)-enabled IoT environment. The rigorous analysis of this paper is worth drawing attention to. However, this approach applies to four types of intrusions only. Moreover, it does not emphasize detection rate optimization. The study by A. Henry et al. [14] follows a similar approach of combining different models. However, it suffers from the same limitation of false alarm rate and detection rate optimization. Similar weaknesses are noticeable in the papers of A. Fatani et al. [15], M. Bacevicius et al. [16], and H. Alshahrani et al. [17]. This paper presents a unique approach to discovering the balance between intrusion detection and false positive rates. It enhances the usability of the IDS and makes it more effective. Similar to other papers, none of these approaches can ensure protection against 14 different types of intrusions.

The study of S. Alosaimi et al. [18] demonstrates an appreciable multi-layer security system using DL for IoT networks. However, it is confined to different types of DDoS attacks only. Moreover, because of using multiple layers, the system has taken on a complicated shape. Compared to this approach, the proposed IDS is simpler yet more efficient. It is a faster and more practical solution to IoT intrusion. The ensemble method presented by Y. Alotaibi et al. [19] deserves appreciation for combining four types of models. However, this effective system does not extend to developing a practical defense mechanism as proposed in this paper. The explainable AI-based approach of X. Larriva-Novo et al. [20] excellently presents the underlying intrusion detection processes. However, the mathematical analysis to justify the selected model is missing in the research discussed in the proposed paper.

The survey on intrusion detection using machine learning by S. V. N. Santhosh Kumar et al. [6], S. Fraihat et al. [21], and B. Kaur et al. [22] supports the hypothesis of this paper. The recent research in intrusion detection using IoT focuses on performance improvement while ignoring the development of application models. Detecting intrusion by training DL networks and applying the model to ensure security is not the same. The latter is more challenging, which has been addressed in the proposed paper. The false positive rate and interruption in IoT response are two major usability drawbacks of IDSs that have been studied and solved in the proposed paper. Moreover, the proposed paper introduces the DAC algorithm that extends the IDS’s ability to defend against further intrusion from the same source. Because of these novelties and outstanding performance, the proposed LSTM-based IDS stands out from the rest.

### Research Gap Analysis

The previous section’s literature review points out the existing research’s limitations. It further compares those limitations with the proposed paper. It demonstrates the existing gap in the current IDS-related research field and how the proposed methodology fills the gaps. These gaps have been listed in Table 1. The most common research gap in every paper reviewed is the direction of the practical application of their proposed methodology. The practical application refers to implementing the IDS in a real IoT network. Most papers developed the IDS and confined their study to intrusion prediction. Developing technology and practically applying it to solve an existing problem is not the same. Most of the papers presented the development of the IDS using various innovative approaches; however, they did not discuss the ways they can be implemented. It has been further observed that the binary classification is where malicious and benign network traffic is detected. This is another research gap. Apart from these two research gaps, some other significant research gaps are specific to different papers. These research gaps have been listed in Table 1 as Significant Weaknesses.

## 3. Methodology

### 3.1. Deep Learning Model Analysis & Selection

Selecting an appropriate network is one of the challenges in DL technology [23]. This statement applies to intrusion detection using DL, as it inherits the natural characteristics of the technology [24]. H. Liu et al. surveyed multiple DL models for intrusion detection [25]. According to this study, Recurrent Neural Networks (RNNs), Long Short-Term Memory (LSTM) networks, and Gated Recurrent Units (GRUs) are more effective than other DL models.

#### 3.1.1. Recurrent Neural Networks (RNNs)

The IoT devices receive short sequential data as network packets. RNNs perform excellently in classifying short sequential data. The working principle of RNNs is defined by Equation (1), where $i_{t}$ is the input at time step *t*; $s_{t}$ is the hidden state; $o_{t}$ is the output; $V_{ss}$, $V_{is}$, and $V_{so}$ are the weight matrices; and $c_{s}$ and $c_{o}$ are the biases. The variable σ(·) is the activation function [26].

(1) $$s_{t}=\sigma \left(V_{ss}s_{t-1}+V_{is}i_{t}+c_{s}\right)$$

(2) $$o_{t}=V_{so}s_{t}+c_{o}$$

According to the mathematical principle of RNN defined in Equations (1) and (2), it is effective at classifying network packets that maintain timestamps. That is why it is a potential candidate for intrusion detection at IoT nodes. However, RNN has limitations, including vanishing gradient problems and performance issues for long sequences.

#### 3.1.2. Long Short-Term Memory (LSTM) Networks

The LSTM network is a variation of RNNs that overcomes the limitations of RNNs [27]. It combines a memory cell to prevent vanishing gradient problems with a tri-gating mechanism to handle long sequences. These gates are defined by Equations (3)–(5), which are input, forget, and output gates, respectively [28].

(3) $$p_{t}=\sigma \left(Q_{p}\left[s_{t-1},i_{t}\right]+d_{p}\right)$$

(4) $$q_{t}=\sigma \left(Q_{q}\left[s_{t-1},i_{t}\right]+d_{q}\right)$$

(5) $$r_{t}=\sigma \left(Q_{r}\left[s_{t-1},i_{t}\right]+d_{r}\right)$$

The input signals to the network update the cell state and specify a candidate cell for the subsequent sequence. As a result, LSTM networks are better at handling network packets. The update and candidate cell states are defined by Equations (6) and (7), respectively.

(6) $${\tilde{D}}_{t}=\tanh\left(Q_{D}\left[s_{t-1},i_{t}\right]+d_{D}\right)$$

(7) $$D_{t}=p_{t}⊙D_{t-1}+q_{t}⊙{\tilde{D}}_{t}$$

The network’s final output is modeled as Equation (8).

(8) $$s_{t}=r_{t}⊙\tanh\left(D_{t}\right)$$

In Equations (3)–(5), $p_{t}$, $q_{t}$, and $r_{t}$ represent the forget, input, and output gates, respectively. $D_{t}$ and ${\tilde{D}}_{t}$ denote the updated and candidate cell states, respectively. The weight matrices $Q_{p}$, $Q_{q}$, $Q_{r}$, and $Q_{D}$ and the bias vectors $d_{p}$, $d_{q}$, $d_{r}$, and $d_{D}$ are the model parameters. The symbol ⊙ denotes element-wise multiplication.

#### 3.1.3. Gated Recurrent Units (GRUs) Network

This is a simplified variant of LSTM networks. Unlike LSTM networks, it uses two gates, updated and reset, expressed as Equations (9) and (10). In Equation (9), $z_{t}$ is the update gate, $W_{z}$ is the weight matrix for the update gate, $h_{t-1}$ is the previous hidden state, $x_{t}$ is the input at the current time step, and $b_{z}$ is the bias for the update gate. And in Equation (10)

(9) $$z_{t}=\sigma \left(W_{z}\cdot \left[h_{t-1},x_{t}\right]+b_{z}\right)$$

(10) $$r_{t}=\sigma \left(W_{r}\cdot \left[h_{t-1},x_{t}\right]+b_{r}\right)$$

The GRU network uses a candidate state, which is a filtered version of the input combined with the previous hidden state. The linear interpolation between the previous and candidate hidden states produces the current hidden state. In Equation (11), ${\tilde{h}}_{t}$ is the candidate hidden state, $W_{h}$ is the weight matrix for the candidate hidden state, and $b_{h}$ is the bias for the candidate hidden state. In Equation (12), $h_{t}$ is the current hidden state.

(11) $${\tilde{h}}_{t}=\tanh\left(W_{h}\cdot \left[r_{t}⊙h_{t-1},x_{t}\right]+b_{h}\right)$$

(12) $$h_{t}=\left(1-z_{t}\right)⊙h_{t-1}+z_{t}⊙{\tilde{h}}_{t}$$

#### 3.1.4. Selection of an Appropriate Deep Learning Model

**Figure 1:**
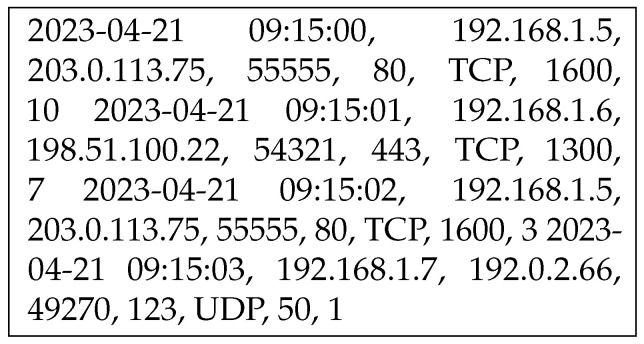
A sample of sequential data in the experimental network.

All three networks mathematically analyzed in Section 3.1 show their ability to handle network sequential data. A sample of the data is presented in Figure 1. To properly detect the intrusion, it is essential to temporarily store the correct state and explore the anomalous nature of the subsequent states. Moreover, the network sequences are random, and the network processing them must be capable of handling both long and short sequences.

Regarding random sequences with varying lengths, the LSTM networks perform better than RNN and GRU networks [29]. Moreover, it is not affected by vanishing gradient problems like RNN. Furthermore, it has three gates capable of adjusting the data flow according to the volume of traffic on the network. Considering the advantages, the LSTM network has been selected as the DL model to develop the IDS.

### 3.2. Dataset & Preprocessing

The CIC-IDS2017 dataset used in this paper is chosen for its comprehensive features, thorough documentation, and widespread recognition. Developed by the Canadian Institute for Cybersecurity, this dataset is consistently updated by the same institution [30]. Comprising over 2.8 million instances, it presents a diverse range of intrusion types in IoT nodes. The dataset encompasses a total of 78 feature variables. Table 2 details a subset representing each class label.

#### 3.2.1. Dataset Cleaning & Splitting

There are multiple missing and duplicate values in the original dataset. They have been manually cleaned. After cleaning, the cleaned dataset has 55,910 instances. This has been split into training, testing, and validation sets with a ratio of 70:15:15. The data have been randomly distributed among these subsets [31]. There are 39,137 instances of training the network at this ratio. Both the testing and validation set have 8386 instances. In every phase of data splitting, the instances have been randomly shuffled. There are 78 exclusive features in the CIC-IDS2017 dataset. It is a labeled dataset with 15 classes. Of these classes, 14 are malicious, and one is benign.

#### 3.2.2. Dataset Normalization

There are multiple numerical features in the dataset whose scale significantly varies. As a result, it is essential to normalize these features to prevent the LSTM network from incorrect prediction. This paper uses the Min–Max algorithm, which performs feature normalization. It maps the target features on a scale of 0 to 1 [32]. It is performed by Equation (13), where the feature value is *x*, and the highest and lowest values of the features are $X_{min}$ and $X_{max}$, respectively.

(13) $$X_{normalized}=\frac{X-X_{min}}{X_{max}-X_{min}}$$

After feature normalization, each feature lies within the 0 to 1 range. As a result, the learning algorithm smoothly converges to the lower error region faster.

#### 3.2.3. Categorical Feature Processing

There are non-numeric features in the CIC-IDS2017 dataset. At the same time, there are some numeric features, for example, IP addresses and port addresses, whose numerical values do not represent themselves. These features have been converted into categorical features using the one-hot encoding scheme. In this process, the categorical values are converted into a vector of zeros, except the index representing the class, which is set to 1. The categorical variable classes $C=\{c_{1},c_{2},...,c_{n}\}$ are represented as $c_{i}$ as defined by Equation (14), where the *i*th index is 1 and the rest are zero [33].

(14) $$\mathrm{one}\_\mathrm{hot}\left(c_{i}\right)=\left[\begin{array}{cccccc} 0 & 0 & \ldots & 1 & \ldots & 0 \end{array}\right]$$

#### 3.2.4. Feature Selection

There are both categorical and numerical features in the dataset. The chi-squared test has been used in this paper to determine the significant association between categorical variables and the target class. The chi-squared statistic is calculated using Equation (15), where $\chi^{2}$ is the chi-squared statistic [34].

(15) $$\chi^{2}=\sum_{i=1}^{r}\sum_{j=1}^{c}\frac{{\left(O_{ij}-E_{ij}\right)}^{2}}{E_{ij}}$$

The chi-squared statistic measures the difference between observed and expected frequencies. If the value is larger, it is evident that the two variables are more dependent. A smaller value of $\chi^{2}$ represents the weak association between two variables [35]. Features that exhibit strong associations with the target variables have been used in this paper.

The numerical features are selected using the variance threshold (VT) method, defined by Equation (16), where *x* is the feature, *n* is the number of instances, and $\bar{x}$ is the mean value of the features. Based on the variance measured using Equation (16), the features are selected, which is expressed by Equation (17).

(16) $$\mathrm{Variance}\left(X\right)=\frac{1}{n}\sum_{i=1}^{n}{\left(x_{i}-\bar{x}\right)}^{2}$$

(17) SelectedFeatures={X|Variance(X)>T}

The significant feature correlation coefficients determined through the chi-squared test conducted in this study are detailed in Table 3. A coefficient threshold of 0.41 was established for this experiment; features with coefficients below this threshold were excluded from consideration in this paper. Applying the 0.41 threshold, the top 20 most impactful features were retained to train the network.

#### 3.2.5. Sequence Generation

The network packets received by the IoT nodes form sequential data with timestamps. However, these sequences vary depending on multiple factors, including the volume of data, encryption algorithm, and inserted malicious code. The proposed LSTM network requires sequential data as the input, which is not readily available in the CIC-IDS2017 dataset. This paper uses the sliding window approach to produce sequential data, which resembles the network packet stream the IoT nodes handle [36]. It has been done using the mathematical principle defined in Equation (18).

(18) $$\left(X_{t-w+1},X_{t-w+2},\cdots ,X_{t}\right)\to Y_{t+1}$$

In Equation (18), the sliding window is represented as *w*, the input feature at timestamp *t* is $X_{t}$, and $Y_{t+1}$ is the output feature at the same timestamp. The window slides over the table and generates input and equivalent output sequences according to the defined timestamp. Because of the data variations in the table, the sequences randomly vary. At the same time, the timestamps are random as well. As a result, it resembles the actual network traffic stream sequence.

### 3.3. IoT Node Architecture and Vulnerabilities

The proposed Intrusion Detector System (IDS) works at the IoT node. Understanding the IoT node architecture and communication protocol is essential for seamlessly integrating the proposed IDS. The IoT node architecture, associated communication protocol, and possible security vulnerabilities have been discussed in this section.

#### 3.3.1. IoT Node Design Pattern

**Figure 2:**
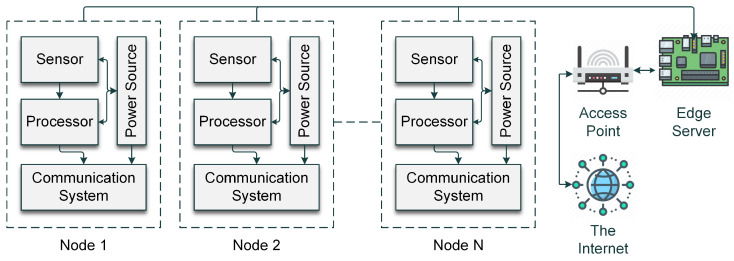
The design pattern of the IoT node.

The IoT nodes connected to the IDS, an edge server, are illustrated in Figure 2. Each node consists of four units. These units are Sensor, Processor, Communication System, and Power Source. The sensors sense the intended input to the IoT device. The processor processes it. The communication system employs the communication protocols and governs the nodes’ communication over the internet. The proposed IDS operates from the edge server at the internet access point (IAP). The edge server communicates with the internet gateway through the IAP [37].

#### 3.3.2. Anomaly Indication Analysis

Malicious signals are injected into the IoT networks, causing IoT devices to deviate from their natural characteristics [38]. It also hampers the regular performance of the devices. The deviation from the natural characteristics and performance issues are prominent indicators of IoT intrusion. Usually, the energy consumption rate changes when an IoT node is compromised [39]. Anomalies in latency and throughput are also indicators of possible intrusion. The energy consumption and throughput are defined by Equations (19) and (20).

(19) $$E=P_{active}\cdot T_{active}+P_{idle}\cdot T_{idle}$$

(20) $$Th=\frac{D}{T}$$

#### 3.3.3. Vulnerabilities & Probabilistic Model

The proposed IDS has been trained on the CIC-IDS2017 dataset. This dataset contains 15 target classes listed in Table 2. There are 14 intrusions and 1 benign class in the target variable. The proposed IDS is capable of detecting these 14 vulnerabilities. A probabilistic model has been developed in this paper, expressed as Equation (21), to define a particular detected intrusion based on its probability [40]. This probabilistic model measures the probability of successful intrusions provided in the dataset. It has been observed that each intrusion on the dataset has the potential to affect the IDS. That is why all 14 intrusions have been considered in this paper.

(21) $$P\left(A|C,S,D\right)=\frac{P(A,C,S,D)}{P(C,S,D)}$$

In Equation (21), P(A) is the probability of a successful attack, and P(C) represents the capabilities of the attacker. The probability of breaching the existing protocol is P(S), and P(D) expresses the probability of the network’s defense mechanisms.

### 3.4. Proposed IDS

There is an LSTM network at the heart of the proposed IDS. It runs from an edge server. The network packets from the internet access the server through the IAP. The bit stream is converted into sequences using the method explained in Section 3.2.5. These sequences are analyzed through the LSTM network. It classifies the sequences and allows them to pass to the IoT node if they are benign. Suppose the sequences carry a malicious signal intended to intrude on the IoT nodes. In that case, they are diverted from them, and necessary actions are taken using the Dynamic Access Control (DAC) algorithm.

#### 3.4.1. LSTM Network Architecture

**Figure 3:**
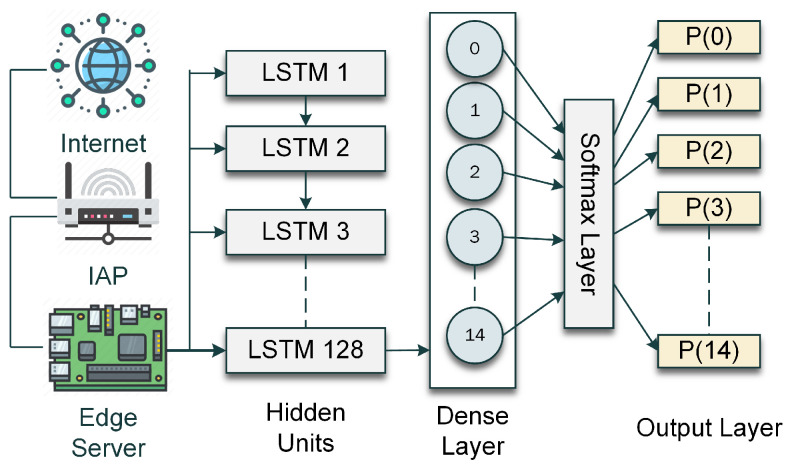
The LSTM network architecture.

The LSTM network designed for the IDS proposed in this paper is illustrated in Figure 3. The architecture of this network has been finalized through multiple iterations by experimenting with different numbers of layers, which have been described in Section 3.4.2. It has been observed that the network performs optimally with 128 LSTM nodes. There is a dense layer after the LSTM layer with 15 nodes. After that, a softmax layer maps the signals from the nodes of the dense layer within the probability scale. Based on this probability, the output layer classifies the input into one of the 15 outputs.

#### 3.4.2. Network Architecture Optimization

**Figure 4:**
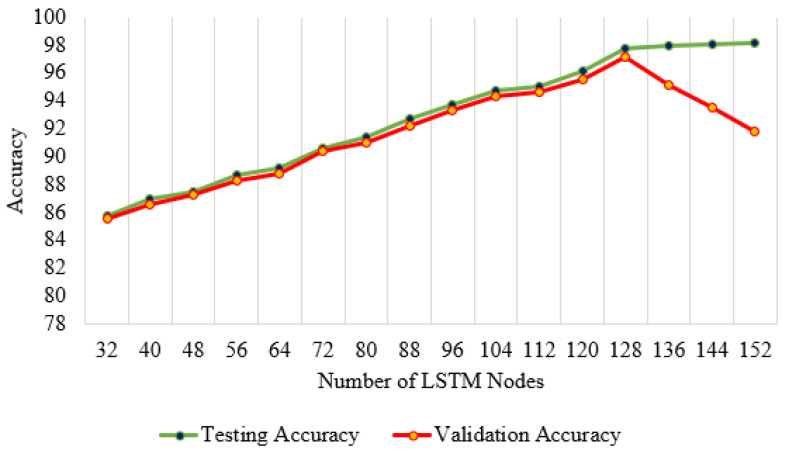
Network architecture optimization through testing and validation accuracy difference.

The network architecture has been optimized through 16 instances of the testing versus validation curve experiment. LSTM nodes increased from 32 to 152 in this experiment, with 8 new nodes simultaneously. The optimized network architecture has been decided from the characteristics and comparison of the testing and validation accuracy curve illustrated in Figure 4.

The difference between training and validation accuracy is marginal. This statement is valid for up to 128 hidden nodes. The training accuracy keeps increasing at a near-constant slope after 128 LSTM nodes. However, the validation accuracy reduces rapidly. This indicates that the network overfits more than 128 LSTM nodes. It also signifies that the network is optimized with 128 LSTM nodes.

#### 3.4.3. Training the Network

**Figure 5:**
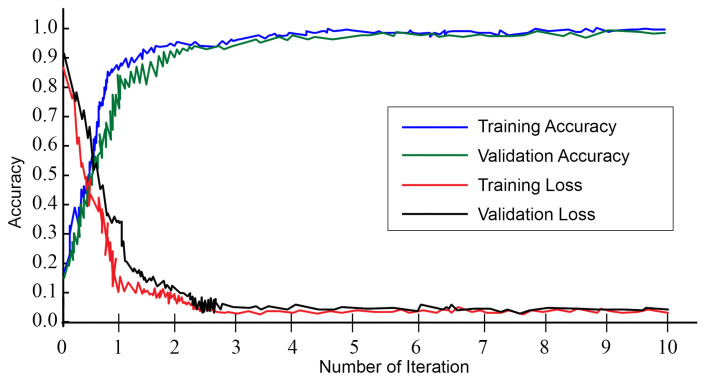
Learning progress with respect to iterations.

The optimized LSTM network has been trained with 39,137 instances. The overall training time is 18 min and 35 s. The learning curves are illustrated in Figure 5. It is evident from the figure that, after the first iteration, the training and validation accuracy maintains consistency. The same statement applies to training and validation loss. The training is completed after 10 epochs with 415 iterations every epoch. The proposed LSTM network learns to classify the intrusions with 97.16% validation and 97.71% training accuracy.

##### Weight Initialization

Random weight initialization is common in deep learning [41]. However, proper weight initialization is key to faster convergence and optimized results [9]. That is why the He weight initialization method has been used in this paper [42]. It is defined by Equation (22), where $N(0,\sqrt{\frac{2}{n_{\mathrm{input}}}})$ is the Gaussian distribution in which the mean is zero, and $\sqrt{\frac{2}{n_{\mathrm{input}}}}$ is the standard deviation.

(22) $$W∼N\left(0,\sqrt{\frac{2}{n_{\mathrm{input}}}}\right)$$

The He weight initialization method generates the primary weights of the nodes from the 0 mean Gaussian distribution and variance. This variance is preserved throughout both back-propagation and forward-propagation. As a result, the probability of the vanishing gradient is lowered. At the same time, it prevents an exploding gradient as the weights maintain a steady variance.

##### Learning Algorithm

The Adaptive Moment Estimation (ADAM) optimization algorithm has been used to train the proposed LSTM network [43]. It combines the Adaptive Gradient (AdaGrad) Algorithm and the Root Mean Square Propagation (RMSProp) Algorithm, so it has both advantages. In the beginning, the first and the second moments are calculated using Equations (23) and (24), respectively. In these equations, the $\alpha_{1}$ and $\alpha_{2}$ represent the exponential decay rates for the moments.

(23) $$p_{t}=\alpha_{1}p_{t-1}+\left(1-\alpha_{1}\right)a_{t}$$

(24) $$q_{t}=\alpha_{2}q_{t-1}+\left(1-\alpha_{2}\right)a_{t}^{2}$$

Once the moments are calculated, it is essential to correct the biases, which has been done using Equations (25) and (26), respectively.

(25) $$\hat{p_{t}}=\frac{p_{t}}{1-\alpha_{1}^{t}}$$

(26) $$\hat{q_{t}}=\frac{q_{t}}{1-\alpha_{2}^{t}}$$

Finally, the weights are updated using Equation (27), where λ is the learning rate, and δ is a small constant. The $\omega_{t}$ in the equation is the updated weight, and $\omega_{t-1}$ is the previous weight [44].

(27) $$\omega_{t}=\omega_{t-1}-\lambda \frac{\hat{p_{t}}}{\sqrt{\hat{q_{t}}}+\delta }$$

### 3.5. Dynamic Access Control (DAC) Algorithm

One of the novel contributions of this paper is the development of the Dynamic Access Control (DAC) algorithm, which is presented as Algorithm 1. This algorithm has been developed to effectively apply the LSTM network developed in this paper to intrusion detection and defense. Furthermore, the algorithm includes additional functionalities to detect intrusion and defend against further intrusion from the same source.

The Dynamic Access Control (DAC) algorithm is presented as Algorithm 1. It senses the presence and absence of network packets. When the bit stream is available, the algorithm enters a while loop and iterates as long as the IDS receives network packets. Initially, the DAC algorithm converts the packet stream into sequences segmented at timestamp *t*. The sequences are then passed to the LSTM network, and the network prediction is stored in variable *d*. If the sequence is not malicious, the algorithm passes the sequence to the IoT node. Otherwise, it dynamically generates a random delay *r* measured in seconds. After that, the IP address of the source is blocked for *r* seconds. At the same time, the packets are discarded, protecting the IoT node. This is how the proposed IDS detects the intrusion and defends against further intrusion from the same source.
**Algorithm 1** Dynamic Access Control Algorithm**Require:** p:NetworkPacket   r←RandBetween(1,2)   **while** p≠0 **do**       $s_{t}\leftarrow \mathrm{Sequence}(p,\;t)$       $d\leftarrow LSTM(s_{t})$       **if** *d* is benign **then**           IoTNode(d)       **else if** *d* is malicious **then**           r←RandBetween(r,r+r)           ip←IPScan(Source(p))           RouterAPI.AccessControl(ip,false,r)           Discard(d)       **end if**   **end while**


## 4. Implementation & Detection Rate Optimization

The proposed methodology has been implemented and experimented with in a test environment. However, the testbed replicates the real-world intrusion pattern, ensuring the experiment’s integrity.

### 4.1. Experimental Setup

The proposed IDS and the DAC algorithm have been hosted in a Raspberry Pi 4 Model B. It has 4 GB primary memory. Odroid N2, NVIDIA Jetson Nano, and Orange Pi 5 are alternate options for the Raspberry Pi 4. The Raspberry Pi 4, with its balance of affordability and performance, is an excellent choice among single-board computers. A vast and active community supports it. While alternatives like the Odroid N2 and NVIDIA Jetson Nano provide higher performance in specific areas, they come at a higher cost and with less community support. The Orange Pi 5, though a strong competitor, still lags in terms of the software ecosystem and user community. The Raspberry Pi 4’s strong support network, software compatibility, and array of compatible accessories make it a highly adaptable and cost-effective option, solidifying its position as the choice to implement the proposed IDS. The Python programming language with the TensorFlow library has been used to implement and train the LSTM network. The DAC algorithm has been coded in Python as well. The Cooja Network Simulator (CNS) [45] has been used to generate IoT network traffic and inject malicious packets [46]. No specific pattern has been maintained while creating the malicious traffic.

### 4.2. Detection Rate Optimization

**Figure 6:**
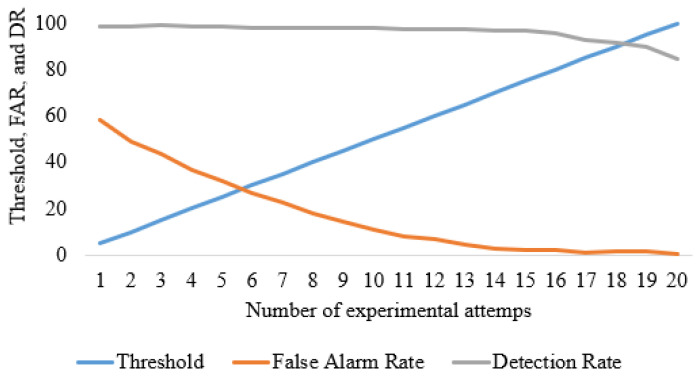
Threshold selection by FAR and DR analysis.

During the experimental observation after implementation, it was observed that the IDS suffers from a high false positive rate (FPR). As a result, the false alarm rate (FAR) is high. It has been further observed that if the detection rate (DR) increases, the FPR increases. Conversely, the FPR declines when the DR declines. The relationship among the DR, FPR, and the threshold is illustrated in Figure 6.

It is observable in Figure 6 that, as the threshold increases, the FPR declines. However, the DR declines as well. Reducing the DR raises a concern about the overall performance of the IDS. On the other hand, high FPR downgrades the effectiveness and usability of the proposed IDS. After in-depth analysis and the trade-off between FPR and DR, it has been observed that the IDS demonstrates optimal performance at 86.88. At this threshold, the FPR is only 1.14%, and the DR is 98.17%.

## 5. Performance Evaluation

The proposed IDS’s performance is analyzed and presented in this section. First, the evaluation metrics are specified in this section, along with their underlying mathematical definitions. After that, a confusion matrix analysis is performed. Finally, the detection rate and response time are analyzed.

### 5.1. Evaluation Metrics

**Figure 7:**
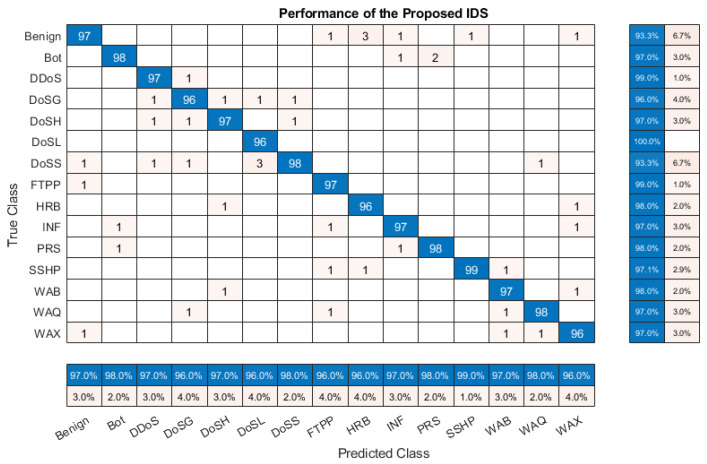
Confusion matrix analysis.

The evaluation metrics observed in the state-of-the-art literature have been used in this paper [47]. These evaluation metrics are derived from the number of instances of true positive (TP), true negative (TN), false positive (FP), and false negative (FN) [48]. These values are obtained from the confusion matrix illustrated in Figure 7. The accuracy, precision, recall (sensitivity), and F1 score have been calculated using TP, TN, FP, and FN. These are defined by Equations (28), (29), (30), and (31), respectively [49].

(28) $$\mathrm{Accuracy}=\frac{\mathrm{TP}+\mathrm{TN}}{\mathrm{TP}+\mathrm{TN}+\mathrm{FP}+\mathrm{FN}}$$

(29) $$\mathrm{Precision}=\frac{\mathrm{TP}}{\mathrm{TP}+\mathrm{FP}}$$

(30) $$\mathrm{Recall}=\frac{\mathrm{TP}}{\mathrm{TP}+\mathrm{FN}}$$

(31) $$F1\;\mathrm{Score}=\frac{2\times (\mathrm{Precision}\times \mathrm{Recall})}{\mathrm{Precision}+\mathrm{Recall}}$$

### 5.2. Confusion Matrix Analysis

The confusion matrix presented in Figure 7 shows the proposed IDS’s performance. Observing the true positive values, the SSHP achieves the highest score of 99, whereas intrusions DoSG, DoSL, HRB, and WAX share the lowest score of 96. Regarding false positives, PRS has the lowest occurrence, with only 1, and DoSH, DoSS, and WAQ have the most with 5. DoSH has the lowest count of 0 for false negatives, while Benign records the highest with 8. The recall percentage is consistently high across all intrusions, ranging from 96% to 99%. The precision metric shows the greatest variation, with DoSL achieving the highest score at 100%, and with Benign and DoSS registering the lowest at 93.3%. The F1 score, which balances precision and recall, falls between 95.1% for Benign and 98% for DDoS, PRS, and SSHP, revealing the overall performance and effectiveness of the model across different intrusions.

Table 4 summarizes the performance of the proposed IDS illustrated in the confusion matrix of Figure 7.

### 5.3. Detection Rate and Response Time Analysis

The proposed IDS’s intrusion detection rate and response time performance are listed in Table 5. The detection rate is measured using Equation (32). The average intrusion attempted is 110, and the average detected intrusion is 108.6. This leads to an average response rate of 1.14 s and an average detection rate of 98.73%.

(32) $$\mathrm{Detection}\;\mathrm{Rate},\;R=\frac{\mathrm{Detected}\;\mathrm{Intrusion}}{\mathrm{Total}\;\mathrm{Intrusion}}\times 100$$

**Figure 8:**
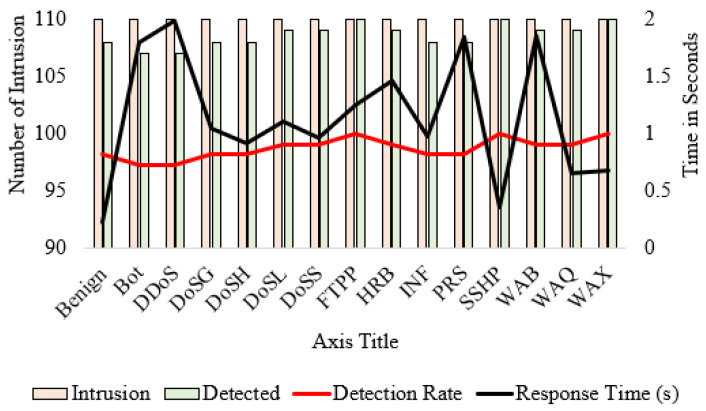
The detection rate and response time.

Noticeably, FTPP, SSHP, and WAX show a perfect detection rate of 100%, with the actual and detected intrusions both at 110. Conversely, Benign, Bot, DDoS, DoSG, DoSH, INF, and PRS recorded slightly lower detection rates at 98.18%, while the remaining intrusions reached 99.09%. Response times exhibit a wider range of values, with SSHP having the fastest response time of 0.36 s and DDoS the longest at 1.98 s. The categories of DoSL and DoSS boast nearly perfect detection, with a moderate response time of 1.1 s and 0.96 s, respectively, balancing efficiency and effectiveness. On the other hand, FTPP, though having a perfect detection rate, takes 1.25 s to respond. The performance of the proposed IDS is illustrated in Figure 8 in terms of detected intrusion, detection rate, and response time.

### 5.4. Performance Comparison

Table 6 showcases a comparative analysis of the proposed methodology’s performance metrics against various other methods from different papers. Each paper here uses a similar data processing method as used in this paper. As a result, this evaluation is not biased towards the processed data of this paper. The metrics assessed include the number of intrusions detected, precision, recall, F1 score, detection rate, and response time. M. A. Almaiah et al. [50] and J. Cui et al. [51] both detect two intrusions, with Almaiah et al. showing a higher precision of 93.94%, as opposed to Cui et al.’s 88.55%. M. Barhoush et al. [52] distinguish themselves by detecting a significantly higher number of intrusions, with a commendable F1 score of 93.9%. However, the proposed methodology outperforms all the listed papers with the same capability of detecting 14 intrusions, boasting an F1 score of 97.05%, and an impressive detection rate of 98.73%. Moreover, it is the only method in the table to report a response time, which is a swift 1.14 s.

### 5.5. Performance on BoT-IoT Dataset

The CIC-IDS2017 dataset is robust and contains a wide range of intrusions. The proposed IDS has been trained with this dataset to extend its detection range. However, another dataset, the BoT-IoT dataset, focuses more on IoT intrusions. An experiment has been conducted to evaluate the performance of the proposed IDS on the BoT-IoT dataset. Even though both CIC-IDS2017 and BoT-IoT datasets contain intrusion-related data, there are some mutually exclusive classes in these two datasets [53]. Similar classes in these two datasets have been identified using Equation (33), where $D_{cic}$ and $D_{bot}$ represent the CIC-IDS2017 and BoT-IoT datasets, respectively.

(33) $$D_{cic}\bar{⊕}D_{bot}=\left\{DoS,DDoS\right\}$$

**Figure 9:**
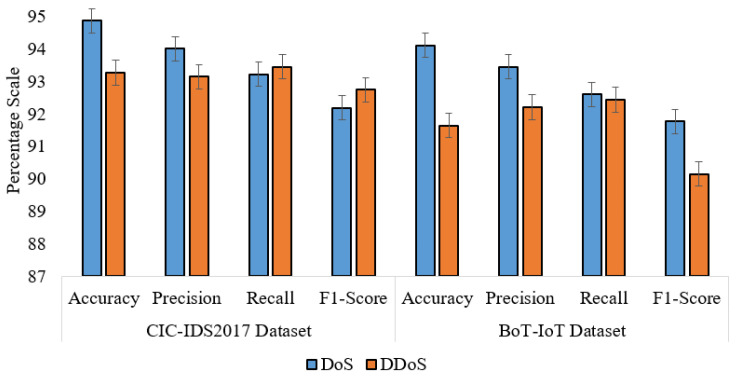
Performance comparison between CIC-IDS2017 and BoT-IoT datasets.

A comparative analysis of proposed IDS performance using the CIC-IDS2017 and BoT-IoT datasets is presented in Table 7. The data reveal a strikingly similar level of effectiveness in detecting both DoS and DDoS attacks across these datasets. For DoS attack detection, the IDS shows an accuracy of 94.87% on the CIC-IDS2017 dataset and a closely comparable 94.11% on the BoT-IoT dataset. This similarity is further mirrored in the precision, recall, and F1 scores, with only minimal differences (less than 1% in most cases). Similarly, for DDoS attacks, the accuracy on CIC-IDS2017 is 93.26%, versus 91.64% on BoT-IoT, again showing a consistent performance level. The precision and recall numbers for DDoS attacks are also quite similar yielding F1 scores close to one another. Such simultaneous performance on two unrelated datasets highlights the effectiveness of IDS, showing its stable efficiency in detection of DoS and DDoS attacks in different network environments. The performance comparison is shown in Figure 9.

### 5.6. Feature Selection Effectiveness

The proposed IDS has been trained with selected features identified through the chi-squared test discussed in Section 3.2.4. As a result, features with a weak correlation with the target variable were excluded from the training process. An experiment has been conducted to analyze the effectiveness of the feature selection method applied in this paper by comparing the training performance of the proposed IDS with and without the feature selection method. Table 8 demonstrates the effectiveness of the feature selection method.

According to the data in Table 8, the convergence time is significantly reduced by 24.69%, from 24.67 min with all features to 18.58 min with selected features, indicating enhanced efficiency. This improvement is paralleled in accuracy metrics; training accuracy sees an increase of 7.29% from 90.42% to 97.71%, and validation accuracy improves by a total of 7.41%, from 89.75% to 97.16%. These figures underscore the substantial benefits of feature selection, not only in reducing computational time but also in significantly enhancing the model’s performance during both training and validation, highlighting its critical role in developing efficient and precise IDS.

### 5.7. Resource Consumption

The hourly resource consumption data of the proposed IDS are listed in Table 9. During the early hours (00:00–03:00), there is a noticeable spike in CPU utilization, particularly at 02:00–03:00, reaching 76%, indicating heightened processing activity. Interestingly, memory usage does not follow a similar trend, remaining relatively stable around 2.5 GB initially and gradually increasing to 3.2 GB by 06:00. Disk I/O operations fluctuate moderately, peaking at 47 IOPS during 05:00–06:00. Network throughput remains consistently below 1.2 Mbps, implying a steady but not overwhelming network load. Power consumption, correlating with CPU usage, escalates from 3 Watts to 4 Watts, suggesting a direct relationship between processing intensity and energy usage.

## 6. Limitations & Future Scope

The performance evaluation of the proposed IDS demonstrates remarkable results. Notwithstanding the outstanding performance, the proposed IDS has multiple limitations, which are discussed in this section.

### 6.1. Implementation Cost

The proposed IDS has been implemented using a Raspberry Pi B headless computer. It is a multipurpose computer and is expensive. As a result, the overall implementation cost of the proposed IDS is high. However, designing an embedded system only for the proposed IDS effectively reduces the cost. Moreover, a faster market adoption would reduce the cost even further. It opens the door to further research on implementing it as an embedded system. It also paves the way for conducting research from a business perspective.

### 6.2. Risk of Adversarial Machine Learning Attack (AML)

The proposed IDS has not been tested with an AML attack. The attempt to analyze the impact of an AML attack failed because of the lack of an appropriate dataset. However, the scope of conducting more experiments remains open. Subsequent research will explore the response to AML attacks and prevention mechanisms for the proposed IDS [33].

### 6.3. Experimental Testbed

The proposed IDS has been studied in an experimental testbed. Every possible measurement has been taken to resemble a real-world intrusion-like behavior of the testbed. However, exploring its performance in a real-world IoT environment would further support the system’s overall integrity. An effort to do so is ongoing. The IDS’s performance in a real-world environment will be presented in future work upon getting the opportunity.

The limitation of the LSTM-based IDS with the DAC algorithm is an opportunity to conduct further research to improve its quality. From this context, these limitations create the opportunity for the future scope of this research.

## 7. Conclusions

The LSTM-based intrusion detection system presented in this paper has an innovative Dynamic Access Control algorithm that detects the intrusion and prevents further intrusions. It blocks the source of intrusion for a dynamic period, which increases according to the number of intrusions. This unique approach makes this paper different than DL-based intrusion detectors. The network architecture has been designed through performance analysis and learning curve deviation. As a result, the LSTM network used in this paper performs optimally. Moreover, the training process used in this paper utilizes the He weight initialization method accompanied by an ADAM optimizer. It contributed to achieving an impressive validation accuracy of 97.16%. The precision, recall, and F1 score of this IDS are 97.07%, 97.11%, and 97.05%, respectively. These numerical evaluations signify the superiority of this IDS. Apart from performance evaluated through machine learning performance evaluation standards, the proposed IDS stands out from the rest because of the analysis from the user perspective. It is a practical solution to IoT intrusion, which detects and defends intrusion within 1.14 s. The detection rate of this IDS has been optimized, and it ensures a minimum number of false alarms. As a result, it does not interrupt the intended service often. The faster response and 1.27% false alarm rate ensure an uninterrupted user experience. Considering the technical and utility perspective, the proposed LSTM-based IDS is one of the most effective solutions to IoT intrusion.

## Figures and Tables

**Table 1:** The research gaps discovered in recent literature reviews.

Author(s)	Method Used	Significant Weaknesses	Practical Application	Detection Range
A. Awajan	Deep Learning	Incapable of interpreting network sequential data	No	5
D. Musleh	Machine Learning	Limited to image features only	No	2
S. Alkadi	Machine Learning	Does not contribute to developing any practical solution	No	4
M. Alazab	Moth–Flame Optimizer (MFO)	Does not discuss effective applications	No	2
R. Chaganti	IDS for SDN-enabled IoT	Limited to four types of intrusions; no focus on detection rate optimization	No	4
A. Henry	Combining Different Models	Lack of false alarm rate and detection rate optimization	No	2
A. Fatani	Not Specified	Similar weaknesses in false alarm rate and detection rate optimization	No	2
M. Bacevicius	Machine Learning	Similar weaknesses in false alarm rate and detection rate optimization	No	2
H. Alshahrani	Not Specified	Similar weaknesses in false alarm rate and detection rate optimization	No	2
S. Alosaimi	Deep Learning	Limited to DDoS attacks; complicated system	No	1
Y. Alotaibi	Ensemble Method	Does not extend to developing a practical defense mechanism	No	2
X. Larriva-Novo	Explainable AI	Lack of mathematical justification	No	2
S. V. N. Santhosh Kumar	Survey Using ML	Focus on performance improvement while ignoring practical applications	No	2

**Table 2:** A subset of the CIC-IDS2017 dataset with all class labels.

Duration	Src IP	Src Port	Dst IP	Dst Port	Protocol	Label
0	10.0.2.15	57158	216.58.208.46	80	6	Benign
5	192.168.10.5	80	192.168.10.50	57164	6	FTP-Patator (FTPP)
4	192.168.10.5	22	192.168.10.50	57165	6	SSH-Patator (SSHP)
3	192.168.10.5	80	192.168.10.50	57166	6	DoS slowloris (DoSS)
2	192.168.10.5	80	192.168.10.50	57167	6	DoS Slowhttptest (DoSL)
1	192.168.10.5	80	192.168.10.50	57168	6	DoS Hulk (DoSH)
0	192.168.10.5	80	192.168.10.50	57169	6	DoS GoldenEye (DoSG)
0	192.168.10.5	443	192.168.10.50	57170	6	Heartbleed (HRB)
3	192.168.10.5	80	192.168.10.50	57171	6	Web Attack—Brute Force (WAB)
2	192.168.10.5	80	192.168.10.50	57172	6	Web Attack—XSS (WAX)
1	192.168.10.5	80	192.168.10.50	57173	6	Web Attack—SQL Injection (WAQ)
0	192.168.10.5	80	192.168.10.50	57174	6	Infiltration (INF)
0	192.168.10.5	80	192.168.10.50	57175	6	Bot
1	192.168.10.5	80	192.168.10.50	57176	6	PortScan (PRS)
0	192.168.10.5	80	192.168.10.50	57177	6	DDoS

**Table 3:** The correlation coefficient according to the chi-squared test.

Feature	Correlation	Feature	Correlation	Feature	Correlation
Flow duration	0.54	Device type	0.57	Payload size	0.27
Number of packets	0.8	Operating system	0.62	HTTP method	0.26
Payload entropy	0.63	Application software	0.52	HTTP request path	0.24
Protocol	0.8	Network topology	0.71	HTTP request headers	0.29
Packet size	0.67	User behavior	0.73	HTTP response code	0.24
TCP flags	0.76	Subnet ID	0.47	HTTP response headers	0.23
IP header flags	0.73	VLAN ID	0.5	IP addresses	0.13
HTTP headers	0.61	Login time	0.41	Device type	0.27
Payload	0.63	Logout time	0.47	Operating system	0.26
Entropy	0.79	Network access patterns	0.46	Application software	0.23

**Table 4:** The performance of the proposed IDS according to the confusion matrix.

Intrusion	TP	FP	FN	Recall	Precision	F1 Score
Benign	97	3	8	97	93.3	95.1
Bot	98	2	3	98	97	97.5
DDoS	97	3	1	97	99	98
DoSG	96	3	4	96	96	96
DoSH	97	5	0	97	97	97
DoSL	96	2	6	96	100	97.9
DoSS	98	4	1	98	93.3	95.6
FTPP	97	4	1	96	99	97.5
HRB	96	3	2	96	98	97
INF	97	2	3	97	97	97
PRS	98	1	3	98	98	98
SSHP	99	3	6	99	97.1	98
WAB	97	2	5	97	98	97.5
WAQ	98	4	3	98	97	97.5
WAX	96	3	3	96	97	96.5

**Table 5:** The detection and the response time of the proposed IDS.

Intrusion	Intrusion	Detected	Response Time (s)	Detection Rate (%)
Benign	110	108	0.23	98.18
Bot	110	107	1.8	97.27
DDoS	110	107	1.98	97.27
DoSG	110	108	1.05	98.18
DoSH	110	108	0.92	98.18
DoSL	110	109	1.1	99.09
DoSS	110	109	0.96	99.09
FTPP	110	110	1.25	100.00
HRB	110	109	1.46	99.09
INF	110	108	0.97	98.18
PRS	110	108	1.84	98.18
SSHP	110	110	0.36	100.00
WAB	110	109	1.85	99.09
WAQ	110	109	0.65	99.09
WAX	110	110	0.68	100.00

**Table 6:** Performance comparison of the proposed methodology with other papers.

Paper	Number of Intrusions	Precision	Recall	F1 Score	Detection Rate
M. A. Almaiah et al.	2	93.94	93.23	94.44	NA
J. Cui et al.	2	88.55	86.59	86.88	NA
M. Barhoush et al.	14	91.77	96.85	93.9	NA
M. Jeyaselvi et al.	2	95.65	92.74	91.48	98.16
N Sarkar et al.	2	88.3	87.78	87.68	NA
Proposed	14	97.07	97.11	97.05	98.73

**Table 7:** Performance evaluation comparison against DoS and DDoS attacks using different datasets.

Intrusion Type	CIC-IDS2017 Dataset				BoT-IoT Dataset			
	**Accuracy**	**Precision**	**Recall**	**F1 Score**	**Accuracy**	**Precision**	**Recall**	**F1 Score**
DoS	94.87	94.01	93.22	92.18	94.11	93.45	92.60	91.77
DDoS	93.26	93.14	93.45	92.74	91.64	92.21	92.44	90.15

**Table 8:** Feature selection method’s effectiveness analysis.

Evaluation Criteria	Selected Features	All Features	Effectiveness
Convergence Time	18.58 min	24.67 min	24.69% less time
Training Accuracy	97.71%	90.42%	7.29% improvement
Validation Accuracy	97.16%	89.75%	7.41% improvement

**Table 9:** Hourly resource consumption of LSTM-based IoT IDS on Raspberry Pi.

Time Interval (Hour)	CPU Utilization (%)	Memory Usage (GB)	Disk I/O (IOPS)	Network Throughput (Mbps)	Power Consumption (Watts)
00:00–01:00	38	2.5	43	0.8	3
01:00–02:00	47	2.3	37	0.7	2.8
02:00–03:00	76	2.1	37	0.9	2.6
03:00–04:00	38	2.8	41	0.9	3.2
04:00–05:00	40	3.0	40	1.0	3.5
05:00–06:00	38	3.2	47	1.1	3.7
06:00–07:00	39	3.5	38	1.2	4.0
07:00–08:00	42	3.8	39	0.9	4.3
08:00–09:00	44	4.0	42	1.2	3.8
09:00–10:00	42	4.2	40	0.9	3.9

## Data Availability

The datasets of this study are available from the corresponding author upon reasonable request.
